# The Impact of Tumor Infiltrating Lymphocytes Densities and Ki67 Index on Residual Breast Cancer Burden following Neoadjuvant Chemotherapy

**DOI:** 10.1155/2022/2597889

**Published:** 2022-09-12

**Authors:** Aya Elmahs, Ghada Mohamed, Mostafa Salem, Dina Omar, Amany Mohamed Helal, Nahed Soliman

**Affiliations:** ^1^Department of Pathology, Faculty of Medicine, Helwan University, Egypt; ^2^Department of Pathology, National Cancer Institute, Cairo University, Egypt; ^3^Department of Pathology, Baheya Center for Early Detection and Treatment of Breast Cancer, Egypt; ^4^Department of Pathology, Faculty of Medicine, Cairo University, Egypt; ^5^Medical Oncology Department, Baheya Center for Early Detection and Treatment of Breast Cancer, Egypt; ^6^Medical Oncology Department, National Cancer Institute, Cairo University, Egypt

## Abstract

To avoid unnecessary neoadjuvant chemotherapy in case anticipating a poor therapy response, it is essential to find the pathological parameters that would predict pathological complete response or at least a decrease in tumor burden following neoadjuvant chemotherapy. The purpose of this study is to investigate the hypothesis that tumor infiltrating lymphocytes can predict the efficacy of neoadjuvant chemotherapy and to find the Ki67 cutoff value that best predicts the benefit of chemotherapy. 153 cases of breast cancer were chosen, based on their molecular subtype: triple negative subtype (77) and luminal, HER2-ve subtype (76). Histopathological assessment of pretherapy core biopsies was conducted to assess variable pathological parameters including TILs rates with the aid of immunohistochemical staining for CD20 and CD3. Moreover, core biopsies were stained for Ki67, and the findings were compared to the residual cancer burden following neoadjuvant chemotherapy. On analyzing and contrasting the two groups, a significant association between molecular subtype and pathological complete response was confirmed, while tumor-infiltrating lymphocytes in either group had no effect on therapy response. We used receiver operating characteristic curve analysis to determine that a cutoff of 36% for Ki67 is the most accurate value to predict complete therapy response.

## 1. Introduction

The primary treatment for advanced breast cancer is neoadjuvant chemotherapy (NACT) [1], that is why identifying the pathological parameters that can anticipate poor therapeutic response can help avoid unnecessary NACT and its accompanying side effects and costs. Despite being the desired target, the pathological complete response (pCR) system is not able to risk-stratify patients with residual cancer following NACT, in contrast to residual cancer burden (RCB) system that can classify patients with residual cancer which has been proven to be of prognostic value [2]. Tumor size, overall cancer cellularity, percentage of in situ carcinoma, number of residually positive lymph nodes, and length of longest nodal metastasis are all factors considered in RCB scoring. Raw scores are then categorized into RCB classes using predefined cut-points, with a score of zero representing pCR and scores from 1 to 3 representing increasing amounts of residual disease [3].

Tumor microenvironment has an important role in the progression of cancer. However, historically breast cancer was considered as immunologically inactive relative to other tumors, particularly melanoma [4]. On the contrary, evidence has emerged that pretherapy tumor infiltrating lymphocytes (TILs) within breast cancer may predict response to therapy [5].

The choice of cutoff points to define high Ki67 levels is controversial. Although most studies suggest that higher grade tumors with a high proliferation index would respond better to NACT [6, 7], the intervariability in manual counting and the controversy of analytical validity decrease the accuracy of ki67 scoring, that is why the limitations of global standardization of Ki67 cutoff value persist [8].

This study is aimed at testing the hypothesis that the presence of a lymphocytic infiltrate in tumor tissue may predict the response to neoadjuvant chemotherapy. Additionally, identifying the optimal Ki67 cutoff point for use as a predictor of chemotherapy benefit. In addition to analyzing which of the studied pathological parameters may aid in predicting therapy response.

## 2. Material and Methods

### 2.1. Study Population

This is a retrospective study that included 153 breast cancer cases, chosen by accessing the database of Baheya Centre for Early Detection and Treatment of Breast Cancer between November 2019 and September 2020. Cases were selected according to their molecular subtype; TNBC (77 cases) and luminal HER2-ve subtype (76 cases). Selected cases should have performed core biopsy followed by NACT with subsequent surgical treatment, whether modified radical mastectomy or quadrantectomy with axillary clearance. Ethics committee approval was granted from the Institutional Review Board of Baheya Centre. Only the principal investigator reviewed the patients' original reports to compile demographic information. A coding number was used instead of a patient's name to identify each case.

Prior to receiving neoadjuvant therapy, all patients should have adequate cell blood count, liver, and kidney functions as well as adequate ejection fraction (≥60%) on echocardiographic evaluation. Details of the treatment regimen were available for the included patients; all patients received a neoadjuvant anthracyclin containing regimen followed by taxanes.

### 2.2. Clinicopathologic Analysis

Tumor core biopsy paraffin blocks were serially sectioned and stained with H&E, and they were evaluated by two qualified pathologists. Tumors were typed according to the WHO histological classification [9]. Grading of the tumors was done using the Nottingham combined grading system [10]. First, H&E-stained slides were used to evaluate the TILs. TILs were then classified into intratumoral lymphocytes (ITLs) and stromal lymphocytes (SLs). ITLs were reported as a percentage of tumor epithelial nests containing infiltrating lymphocytes and defined as intraepithelial mononuclear cells within tumor cell nests or in direct contact with tumor cells. While SLs were defined as the percentage of tumor stroma area that contains a lymphocytic infiltrate without direct contact with tumor cells [11]. There was no consideration given to the immune infiltrate surrounding DCIS or normal breast lobules, neither areas with crush artefacts and necrosis [11, 12]. Tumors were quantitively divided into three groups based on the expression of SLs and/or ITLs, namely, lymphocyte poor tumors (0-10%), intermediate group (20-40%), and lymphocyte predominant tumors (50-90%) [12]. Cores were immunostained with CD3 (rabbit monoclonal antibody), CD20 (mouse monoclonal antibody), and Ki67 (rabbit monoclonal antibody). All markers were ready to use and are obtained from Cell Marque, Sigma-Aldrich, Missouri, United States. BENCHMARK XT IHC/ISH staining module (Ventana) was used for immunostaining.

### 2.3. Assessment of RCB

Following surgery, data was taken from the reports, and slides were reviewed to collect the following parameters: tumor bed size, residual tumor cellularity, percentage of ductal carcinoma that is in situ, number of positive lymph nodes, and size of the largest tumor metastasis.

RCB class was calculated using the MD Anderson Residual cancer burden calculator http://www3.mdanderson.org/app/medcalc/index.cfm?pagename=jsconvert3.

### 2.4. Statistical Analysis

The statistical package for the social sciences (SPSS) version 26 (IBM Corp., Armonk, NY, USA) was used to code data and statistical analysis. Kolmogorov-Smirnov test (KS-test) was used to evaluate the data which was found to be nonparametric [13]. Mean, standard deviation, median, minimum, and maximum values were used to summarize quantitative data, while frequency (count) and percentage were used to summarize categorical data. Comparing quantitative variables was done using the Kruskal-Wallis and Mann–Whitney nonparametric tests. The chi-square test was used to compare categorical data. However, Fisher exact test was used when the expected frequency was less than 5. The Spearman correlation coefficient was used to calculate correlations between quantitative variables. To test whether the link between triple negative subtype and pCR is independent of TILs, we used logistic regression analysis that was performed. And, to study the effect of all positive predictors on pCR, multiple logistic regression analysis was also used. To find the best cutoff value of Ki67 for the detection of pCR, a ROC curve was constructed using the area under the curve analysis. Statistical significance was defined as a *p* value lower than 0.05.

## 3. Results

**Figure 1:**
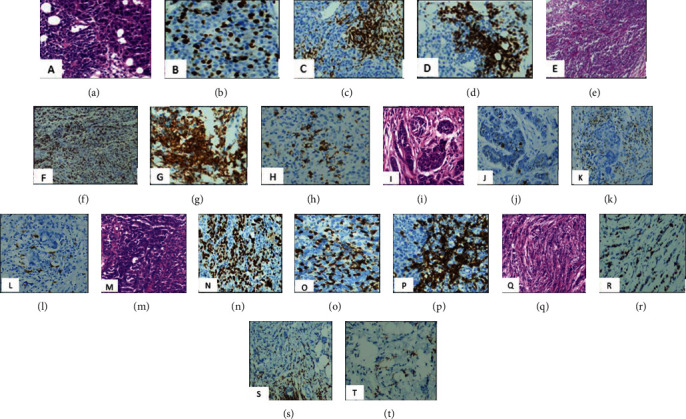
(a–d) Case (1) IDC, grade 3 (lymphocyte predominant); 10% ITL, 70% SL (H&E 40×), (b) Ki67 IHC (40×) proliferation index 40%, (c) CD3 IHC (40×), 60%, (d) CD20 IHC (40×), 40%. (e–h) Case [2] IDC, grade 3 (intermediate group); 20% ITL, 40% SL (H&E) (10×), (f) Ki67 IHC (10×) proliferation index 60%, (g) CD3 IHC (40×), 60%, (h) CD20 IHC (40×), 20%. (i–l) Case [3] IDC, grade 2 (lymphocyte poor); 5% ITL, 10% SL (H&E (40×), (j) Ki67 IHC (40×) proliferation index 5%, (k) CD3 IHC (40×), 70%, (l) CD20 IHC (40×), 10%. (m–p) Case [4] Medullary carcinoma, grade 3 (lymphocyte predominant); 30% I1L, 80% SL (H&E (40×), (n) Ki67 IHC (40×) proliferation index 90%, (o) CD3 IHC (40×), 60%, (p) CD20 IHC (40×), 30%. (q–t) Case [5] ILC, grade 2 (intermediate group); 5% ITL, 40% SL (H&E 10×), (r) Ki67 IHC (40×) proliferation index 50%, (s) CD3 IHC (40×), 50%, (t) CD20 IHC (40×), 5%.

A total of 153 cases were included in the analysis. All patients were female with median age 48 years (range = 28 − 76 years). Patient characteristics, clinicopathological features, and results of immunohistochemical analysis for the entire study population are shown in (Table 1). RCB classes I, II, and III were attended in 3.3%, 32.7%, and 45.1% of the cases, while pCR was achieved in 19% of study cases (Table 1). By using the predefined cut points, lymphocyte poor, intermediate group, and lymphocyte predominant were attended in 17.6%, 43.1%, and 39.2% of study cases (Figure 1; case [1–5] A, E, I, M, and Q).

### 3.1. Relation between Different Pathological Features and RCB Classes

There was no significant association between tumor histological type and the RCB classes nor with pCR. In invasive duct carcinoma, pCR, RCB-I, RCB-II, and RCB-III were achieved in 18.7%, 3.3%, 35.8%, and 42.3% of cases, respectively. Tumor grade shows significant association with both RCB classes and pCR (*p* value < 0.001 and 0.008, respectively). The presence of lymphovascular invasion (LVI) was significantly associated with RCB classes and pCR (*p* value < 0.001, each). Molecular subtype was significantly associated with both pCR and decreased tumor burden (*p* value < 0.001, each) with pCR and RCB-I attended in 35 and 5.2%, respectively, in TNBC in comparison to 2.6% and 1.3%, respectively, in Luminal subtype (Table 2).

Regarding TILs, there was no significant association with RCB nor with pCR (*p* value = 0.623 and 0.147, respectively) (Table 2). By using of the predefined cut points, lymphocytic predominant tumors achieved pCR, RCB-I, RCB-II, and RCB-III in 26.7%, 3.3%, 31.7%, and 38.3% of the cases, respectively. Intermediate group achieves pCR, RCB-I RCB-II, and RCB-III in 13.6%, 3%, 33.3%, and 50% of cases, respectively, while the lymphocyte poor group achieves PCR, RCB-I, RCB-II, and RCB-III in 14.8%, 3.7%, 33.3%, and 48.1% of cases, respectively. When further classifying cases according to the molecular subtype, TILs fail to show significant association with pCR in both luminal and TN subtypes (*p* value = 1 and 0.803, respectively) (Table 3). By logistic regression analysis, the absence of a statistically significant association between TILs and pCR was confirmed despite the molecular subtype (Table 4).

**Figure 2:**
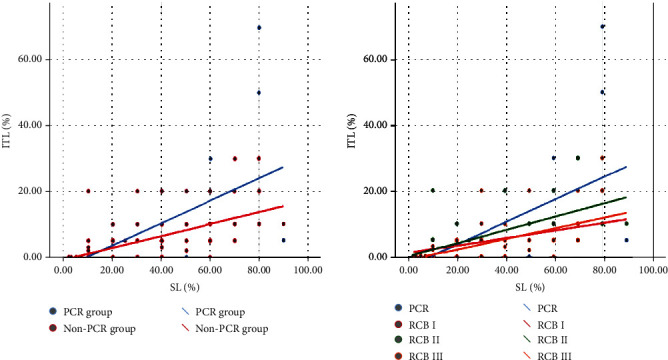
Correlation between ITL and SL using Spearman correlation coefficient.

CD20 and CD3 range from (0-60%) to (2-95%), respectively, among study cases (Figure 1; case [1–5] C, D, G, H, K, L, O, P, S, and T). There was no significant relation between CD20 B lymphocytes and RCB classes (*p* value = 0.735), nor with pCR (*p* value = 0.673). Similarly, CD3 T lymphocytes show no significant difference neither with RCB classes nor with pCR (*p* value = 0.701 and 0.534, respectively (Table 5). Regarding the types of TILs, Spearman correlation proved the positive correlation between SL and ITL (Figure 2).

**Figure 3:**
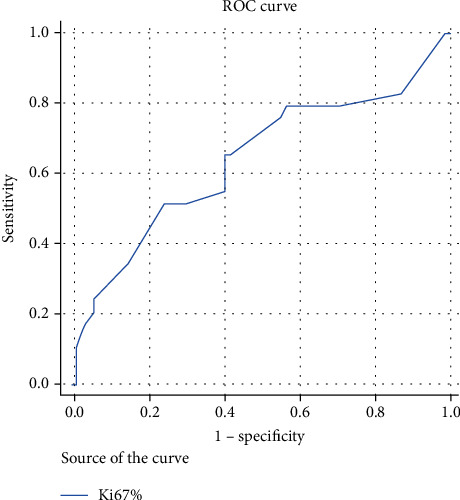
ROC curve analysis to define the cut of point of Ki67%.

Ki67 index ranges from 2% up to 98% (Figure 1; case (1–5) B, F, J, N, and R). Ki67 proliferation index showed a significant relation with both RCB class and pCR (*p* value = 0.002 and 0.018, respectively). Ki67 is expressed by median value of 60%, 60%, 40%, and 20% in RCB-0, RCB-I, RCB-II, and RCB-III, respectively (Table 5). And by ROC curve analysis, we identified 36% as the optimal Ki67 cut-off value to predict the pathological complete response (Figure 3).

**Figure 4:**
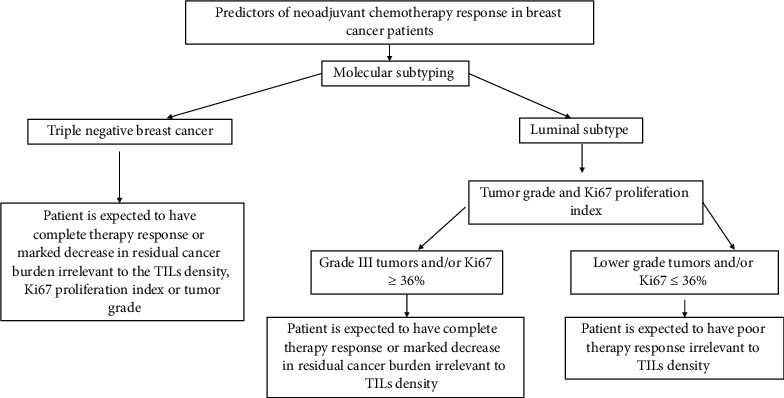
A guidance plan to define whether a breast cancer patient is candidate for neoadjuvant chemotherapy or not.

To define the predictive power of each variable regarding therapy response, logistic regression analysis was performed for molecular subtype, Ki67, LVI, and tumor grade; and it shows that molecular subtype and negative LVI have the upper hand when predicting therapy response (Table 6 and Figure 4).

## 4. Discussion

Two nearly equal groups of two molecular subtypes were analyzed in this study: (luminal, HER2 negative) and TNBC. By using this subtype selection-based approach, we were able to investigate whether the lymphocytic infiltrate's predictive role is restricted to a specific molecular subtype [11, 14]. We have classified TIL into three groups: lymphocyte predominant, intermediate, and lymphocyte poor [4].

### 4.1. Relation between Histological Types, Tumor Grades, LVI, and NAC Response

In terms of correlation with the included pathological parameters, we found no difference between RCB and pCR systems. In our study, we found no association between tumor type and pCR nor RCB classes, as did Nagao and his colleagues [15]. However, due to sample size limitations for rare histological subtypes in the mentioned study as well as our own research, larger studies are required to assess the impact of neoadjuvant chemotherapy on various breast cancer subtypes.

There was a significant association between higher tumor grade and both pCR and lower tumor burden (*p* value = 0.001∗ and 0.008∗). Many previous studies had found similar results [16, 17]. That is why assessment of histological grade has always considered as an important factor in the prognosis of breast cancer patients and should be incorporated into staging systems and treatment algorithms [18].

According to the results of this study, NACT had a significantly worse outcome in patients who had LVI. Previously, similar findings were found [19]. Hence, routine assessment of LVI is linked to good tumor tissue assessment practice [20].

### 4.2. Relation between Luminal Subtypes, TILs, and NAC Response

Following NACT, pCR was observed in 19% of the study cases. Similar results were found in previous studies; 19.2%, 15.2%, and 12% in [16, 21, 22]. Triple-negative subgroup attended pCR in 35.1% of the cases. This was in line with the global average of pCR for triple-negative breast cancer as numerous studies have confirmed these findings [16, 23]. For luminal tumors, a pCR rate of 2.6% was obtained. Previous study revealed a variable but close rates [24]. This result was also consistent with what was mentioned by Torrisi and his colleges, with an expected range for pCR in luminal tumors being 0-18% [25]; as a result of low pCR rate after NACT, hormone therapy is still being used as a primary treatment for hormonal positive tumors worldwide after NACT [26]. A more recent study suggests that better outcomes can be achieved by excluding hormonal positive tumors from NAC planning [27].

Upon categorizing posttherapy residual cancer into RCB classes, luminal and HER2-ve tumors were more likely to be categorized as RCB-III and II (68.4% and 27.6%), respectively, whereas post-NAC TNBC was more likely to be RCB-0 (35.1%) (*p* < 0.001). The same results were reported by Hamy and his colleagues [28]. That is why, despite the fact that TNBC has a higher recurrence rate and a lower survival rate than other breast cancer subtypes, it is significantly more responsive to chemotherapy [29].

For luminal-HER2 negative included cases, TILs do not appear to have a significant correlation with pCR, as has been found in many previous studies [30, 31] and in a meta-analysis performed by Gao and his colleagues [32]. In contrary to the findings of most of the reviewed studies, we were unable to find a significant association between TILs and pCR in TNBC [33, 34]. With more comprehensive review, these studies have never performed a linearity test for this analysis [33, 34], unlike Hamy et al.'s study that detected a significant but nonlinear relationship between TILs and pCR in TNBC [35]. Neither of these studies have been able to explain why pre-NACT TILs were exclusively linked to pCR in TNBC [33, 34]. A prominent inflammatory infiltrate is a well-known feature of TNBC [36, 37]. To confirm whether the observed significance was due to the molecular subtype itself or it is linked to the density of TILs, we used logistic regression and a significant association between pCR, and TNBC subtype was confirmed irrelevant to TILs. As a result, Laenkholm and his colleagues advised caution when using TILs as a prognostic factor, as well as combining it with other well-established prognostic factors like tumor size and lymph node status, as well as performing the assessment in conjunction with PD-L1 IHC [38].

Further to that, more research studies are needed on tumor biology and cross-talk between immune system components and tumor cells as those can be the main determinants of therapy response. Additionally, the interactions between TILs and chemotherapy regimen may play an important role in this context.

### 4.3. SLs and ITLs

In this research, we discovered a strong correlation between SL and ITL (*p* value < 0.001). That is consistent with the findings mentioned by Denkert and his colleagues [11]. An explanation for this could be found in the initial hypothesis that the static situation of TILs in histologic tissue may be artificial, because lymphocytes are free to move within the microenvironment of a living tissue [30].

In this study, primarily CD3 T cells and then CD20 B cells are found in the tumor microenvironmet, which is consistent with most previous studies [27, 28]. Our study found no correlation between CD20 B-cells and NACT response nor RCB classes. In regard to the relationship between tumor-infiltrating CD20 B cells and neoadjuvant chemotherapy, previous studies were inconclusive [29, 30]. Some studies found that the mean of CD20 B cells in tumors was not significantly associated with chemotherapy effect [31, 32]. However, a link has been found between B cell infiltration and poor prognosis in a recent clinical study [39]. It is important to note that because CD20 B cells have lower levels than T lymphocytes, little is known about their role in the tumor microenvironment and the outcome of therapy. More research with larger sample sizes is needed to clarify this point.

CD3 T-cells had no effect on pCR nor RCB classes of cancer. In a previous study, Mao and his colleagues found that CD3 had no predictive value, which is consistent with these findings [40], while CD3 T-cells and pCR have been shown to be linked in other studies [34, 41]. This study's findings may be explained by what Kolesta said about the redundancy of lymphocytic markers in the prognosis of breast cancer. If we look at the biology of the tumor immune contexture and the endless list of technical and methodology issues, this redundancy could be accepted [42]. Despite Kolesta's conclusion, and due to the small sample size of our study and Mao's study, our recommendation is that the results of this analysis should be interpreted cautiously.

### 4.4. Relation between Ki67 Proliferation Index and NAC Response

A greater likelihood of pCR following NACT was associated with higher Ki67 levels in the current study. According to previous researches, the same findings were found [7, 43]. This was expected, as measures of tumor cell proliferation are typically associated with a greater likelihood of pCR [6].

The median value for Ki67 in our study was 30% with a range of (2-98%). To test human visual perception, we did not use image analysis in our work; instead, we preferred the pathologist's “trained eyes” because this aligns with what pathologists do on in their routine work. By using the ROC curve analysis, the optimal cutoff for Ki67 to predict pCR was 36%. This is in agreement with Denkert and his colleague's findings which mentioned Ki67 > 35% to be an optimal threshold for pCR [44].

Our work aligns with the recommendations of the International Ki67 Breast Cancer Working Group (IKWG) regarding Ki67 IHC being accepted to decide chemotherapy for the patient if it has a value of 30% or more after maintaining training modules for pathologists together with adopting good staining and scoring protocols [43].

Finally, in this study and based on the logistic regression analysis, we were able to identify the predictors of NAC response with molecular subtyping and negative LVI being the most important predictors followed by high tumor grade and high Ki6 > 36%.

### 4.5. Conclusion

TNBC, negative LVI, higher tumor grade, and a Ki67 proliferation index ≥ 36% were found to be predictors of pCR or at least reduction in residual cancer burden following NACT. With further research, we may be able to develop a new scoring system that considers both clinical and pathological parameters when predicting how patients will respond to NACT.

## Figures and Tables

**Table 1:** Distribution of demographic data, pathological features collected from pretherapy core biopsies and parameters investigated in the posttherapy specimens.

	Count (%)
Laterality	
Rt	69 (45.1%)
Lt	84 (54.9%)
Histological type	
IDC+	123 (80.4%)
Non-IDC	30 (19.6%)
Histological type details	
IDC	123 (80.4%)
ILC	11 (7.2%)
Medullary	9 (5.9%)
Metaplastic	5 (3.3%)
Tubulolobular	2 (1.3%)
Grade	
I	6 (3.9%)
II	86 (56.2%)
III	61 (39.9%)
LVI+	
Yes	89 (58.2%)
No	64 (41.8%)
M. subtype+	
Luminal	76 (49.7%)
TNBC	77 (50.3%)
Lymphocyte groups	
Lymphocyte poor	27 (17.6%)
Lymphocyte intermediate	66 (43.1%)
Lymphocyte predominant	60 (39.2%)
Post-neoadjuvant T stage+	
T0	36 (23.5%)
T1	60 (39.2%)
T2	51 (33.3%)
T3	5 (3.3%)
T4	1 (0.7%)
N stage+	
N0	66 (43.1%)
N1	34 (22.2%)
N2	31 (20.3%)
N3	22 (14.4%)
Postneoadjuvant RCB	
RCB 0	29 (19.0%)
RCB I	5 (3.3%)
RCB II	50 (32.7%)
RCB III	69 (45.1%)
Postneoadjuvant pCR groups	
PCR group	29 (19.0%)
Non-PCR group	124 (81.0%)

+IDC: invasive duct carcinoma; +ILC: invasive lobular carcinoma; +LVI: lymphovascular invasion; M. subtype: molecular subtype; N stage: nodal stage; +pCR: pathological complete response; RCB: residual cancer burden; T stage: tumor stage; +TNBC: triple negative breast cancer.

**Table 2:** Association (count (%)) between various parameters of 153 breast cancer cases and RCB classes (chi-square test).

Breast cancer parameters	RCB				*p* value
	PCR	Non-PCR groups			
	RCB 0	RCB I	RCB II	RCB III	
	Count (%)	Count (%)	Count (%)	Count (%)	
Type					
IDC+	23 (18.7%)	4 (3.3%)	44 (35.8%)	52 (42.3%)	0.340
Non-IDC	6 (20.0%)	1 (3.3%)	6 (20.0%)	17 (56.7%)	
Grade					
I	0 (0.0%)	0 (0.0%)	3 (50.0%)	3 (50.0%)	<0.001∗
II	10 (11.6%)	3 (3.5%)	21 (24.4%)	52 (60.5%)	
III	19 (31.1%)	2 (3.3%)	26 (42.6%)	14 (23.0%)	
LVI+					
Yes	1 (1.1%)	0 (0.0%)	21 (23.6%)	67 (75.3%)	<0.001∗
No	28 (43.8%)	5 (7.8%)	29 (45.3%)	2 (3.1%)	
M. subtype+					
Luminal	2 (2.6%)	1 (1.3%)	21 (27.6%)	52 (68.4%)	<0.001∗
TNBC+	27 (35.1%)	4 (5.2%)	29 (37.7%)	17 (22.1%)	
Lymphocyte groups					
Lymphocyte poor	4 (14.8%)	1 (3.7%)	9 (33.3%)	13 (48.1%)	0.623
Intermediate group	9 (13.6%)	2 (3.0%)	22 (33.3%)	33 (50.0%)	
Lymphocytic predominant	16 (26.7%)	2 (3.3%)	19 (31.7%)	23 (38.3%)	

∗ denotes statistically significant. +IDC: invasive duct carcinoma; +LVI: lymphovascular invasion; +M. subtype: molecular subtype; +TNBC: triple negative breast cancer.

**Table 3:** Association count (%) between TIL and PCR in the 2 molecular subtypes among 153 breast cancer cases (chi-square test).

Luminal, HER2-ve subtype	Posttherapy groups		*p* value
	PCR group	Non-PCR group	
	Count (%)	Count (%)	
Lymphocyte groups			
Lymphocyte poor	0 (0.0%)	16 (21.6%)	1
Intermediate group	1 (50.0%)	38 (51.4%)	
Lymphocytic predominant	1 (50.0%)	20 (27.0%)	
TN+ subtype			
Lymphocyte groups			
Lymphocyte poor	4 (14.8%)	7 (14.0%)	0.803
Intermediate group	8 (29.6%)	19 (38.0%)	
Lymphocytic predominant	15 (55.6%)	24 (48.0%)	

∗ denotes statistically significant. + TNBC: triple negative subtype.

**Table 4:** Logistic regression to analyze role of molecular subtype.

	*p* value	OR	95% C.I	
			Lower	Upper
PCR				
Lymphocyte groups	0.711			
Lymphocyte groups (intermediate group)	0.866	0.886	0.219	3.583
Lymphocyte groups (lymphocytic predominant)	0.673	1.331	0.353	5.015
M. subtype+ (TN+)	<0.001∗	18.515	4.169	82.217

∗ denotes statistically significant. +M. subtype: molecular subtype; +TNBC: triple negative subtype.

**Table 5:** Relation between quantitative parameters and RCB classes (Mann–Whitney test).

	RCB				*p* value
	PCR	Non-pCR			
	PCR	RCB I	RCB II	RCB III	
	Median (min-max)	Median (min-max)	Median (min-max)	Median (min-max)	
Ki67%	60 (5.00-95.00)	60 (2-90)	40 (3-98)	20 (5-80)	0.002∗
CD3 T cells %	60 (40-90)	80 (40-80)	60 (2-95)	70 (10-90)	0.701
CD20 B cells %	10 (0-60)	10 (2-20)	20 (0-50)	20 (0- 55)	0.735

∗ denotes statistically significant.

**Table 6:** Logistic regression analysis defines triple negative subtype and negative lymphovascular invasion as best predictors of pCR.

	*p* value	OR	95% C.I.	
			Lower	Upper
PCR				
M. subtype (TNBC)	0.025∗	6.189	1.262	30.343
Negative LVI	0.001∗	35.392	4.458	281.000

∗ denotes statistically significant. +LVI: lymphovascular invasion; +TNBC: triple negative breast cancer.

## Data Availability

Raw data were collected by accessing the database of Baheya Centre for Early Detection and Treatment of Breast Cancer. Derived data supporting the findings of this study are available from the corresponding author on request.

## Abbreviations

DCIS: Ductal carcinoma in situ
IKWG: International Ki67 Breast Cancer Working Group
ITLs: Intratumoral lymphocytes
IHC: Immunohistochemical staining
KS: Kolmogorov-Smirnov test
LVI: Lymphovascular invasion
NACT: Neoadjuvant chemotherapy
pCR: Pathological complete response
ROC: Receiver operating characteristic
RCB: Residual cancer burden
SPSS: Statistical package for the social sciences
SLs: Stromal lymphocytes
TNBC: Triple negative breast cancer
TILs: Tumor infiltrating lymphocytes.

## Supplementary Materials

Supplementary Materials Table 1: distribution of demographic data, pathological features collected from pretherapy core biopsies and parameters investigated in the posttherapy specimens. Table 2: relation between categorical parameters in 153 breast cases and pCR (chi-square test). Table 3: relation between quantitative parameters in 153 breast cases and pCR (Mann–Whitney test).
