# Supplementary material for: The Impact of Tumor Infiltrating Lymphocytes Densities and Ki67 Index on Residual Breast Cancer Burden following Neoadjuvant Chemotherapy
Source: Int J Breast Cancer. 2022 Sep 12;2022:2597889. doi: 10.1155/2022/2597889 (PMC9484975; doi:10.1155/2022/2597889)
Supplement: Supplementary Materials — Table 1: distribution of demographic data, pathological features collected from pretherapy core biopsies and parameters investigated in the posttherapy specimens. Table 2: relation between categorical parameters in 153 breast cases and pCR (chi-square test). Table 3: relation between quantitative parameters in 153 breast cases and pCR (Mann–Whitney test). [file 2597889.f1.docx]

**Supplementary material:**

TABLE 1: Relation between categorical parameters in 153 breast cases and pCR (chi square test)

|  | | **pCR group** | **Non-pCR group** | **P value** |
| --- | --- | --- | --- | --- |
|  |  | **Count (%)** | **Count (%)** |  |
| **Type** | **IDC** | 23 (18.7%) | 100 (81.3%) | 0.879 |
|  | **Non-IDC** | 6  (20.0%) | 24  (80.0%) |  |
| **Grade** | **I** | 0  (0.0%) | 6 (100.0%) | 0.008* |
|  | **II** | 10  (11.6%) | 76 (88.4%) |  |
|  | **III** | 19  (31.1%) | 42  (68.9%) |  |
| **LVI** | **Yes** | 1  (1.1%) | 88 (98.9%) | < 0.001* |
|  | **No** | 28  (43.8%) | 36 (56.3%) |  |
| **M. subtype** | **Luminal, HER 2 -ve** | 2 (2.6%) | 74 (97.4%) | < 0.001* |
|  | **TNBC** | 27 (35.1%) | 50 (64.9%) |  |
| **lymphocyte groups** | **Lymphocyte poor** | 4  (14.8%) | 23  (85.2%) | 0.147 |
|  | **Intermediate group** | 9 (13.6%) | 57  (86.4%) |  |
|  | **Lymphocytic predominant** | 16 (26.7%) | 44  (73.3%) |  |

+IDC: Invasive duct carcinoma, +ILC: Invasive lobular carcinoma, +LVI: Lymphovascular invasion, +TNBC: Triple negative breast cancer

TABLE 2: Relation between quantitative parameters in 153 breast cases and pCR (Mann-Whitney test)

|  |  |  |  |
| --- | --- | --- | --- |
|  | **pCR** | **Non-pCR** |  |
|  | **Median (min-max)** | **Median (min-max)** |  |
| **KI67 %** | 60.00 (5.00-95.00) | 30.00 (2.00-98.00) | 0.011* |
| **CD3 T cells %** | 60.00 (40.00-90.00) | 70.00 (2.00-95.00) | 0.534 |
| **CD20 B cells %** | 10.00 (0.00-60.00) | 20.00 (0.00-55.00) | 0.673 |
